# (1*E*,3*E*)-1,4-Bis(4-meth­oxy­phen­yl)buta-1,3-diene

**DOI:** 10.1107/S1600536810037141

**Published:** 2010-09-30

**Authors:** Gopinathan Narayan, Nigam P. Rath, Suresh Das

**Affiliations:** a Photosciences and Photonics Section, Chemical Sciences and Technology Division, National Institute for Interdisciplinary Science and Technology, CSIR, Trivandrum, Kerala 695 019, India; b Department of Chemistry and Biochemistry and Center for Nanoscience, University of Missouri-St. Louis, One University Boulevard, St. Louis, MO 63121-4400, USA

## Abstract

The title compound, C_18_H_18_O_2_, which exhibits blue emission in the solid state, is an inter­mediate in the preparation of liquid crystals and polymers. The mol­ecule is located on an inversion centre. In the crystal, mol­ecules are arranged in a herringbone motif.

## Related literature

For related structures, see: George *et al.* (1998 ▶); Vishnumurthy *et al.* (2002 ▶); Davis *et al.* (2004 ▶, 2008 ▶); Kumar *et al.* (2009 ▶); Ono *et al.* (2009 ▶). For the synthesis and the use of the title compound in the preparation of polymers and chiral liquid crystals, see: Rotarski (1908 ▶); Wang *et al.* (2003 ▶); Das *et al.* (2008 ▶). For mol­ecules with a herringbone arrangement, see: Koren *et al.* (2003 ▶).
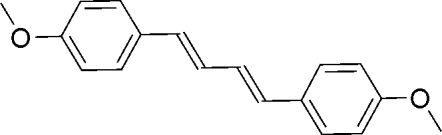


## Experimental

### 

#### Crystal data

C_18_H_18_O_2_
*M* *_r_* = 266.32
Orthorhombic, 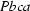

*a* = 7.3543 (3) Å
*b* = 6.2617 (3) Å
*c* = 31.3872 (13) Å
*V* = 1445.39 (11) Å^3^
*Z* = 4
Mo *K*α radiation
μ = 0.08 mm^−1^
*T* = 293 K
0.25 × 0.22 × 0.22 mm

#### Data collection

Bruker X8 APEXII CCD area-detector diffractometer
Absorption correction: numerical (*SADABS*; Sheldrick, 2006 ▶) *T*_min_ = 0.981, *T*_max_ = 0.983
40427 measured reflections
1658 independent reflections
1287 reflections with *I* > 2σ(*I*)
*R*_int_ = 0.036

#### Refinement

*R*[*F* ^2^ > 2σ(*F* ^2^)] = 0.045
*wR*(*F* ^2^) = 0.110
*S* = 1.08
1658 reflections
92 parameters
H-atom parameters constrained
Δρ_max_ = 0.13 e Å^−3^
Δρ_min_ = −0.13 e Å^−3^

### 

Data collection: *APEX2* (Bruker, 2001 ▶); cell refinement: *SAINT* (Bruker, 2001 ▶); data reduction: *SAINT*; program(s) used to solve structure: *SHELXS97* (Sheldrick, 2008 ▶); program(s) used to refine structure: *SHELXL97* (Sheldrick, 2008 ▶); molecular graphics: *SHELXTL* (Sheldrick, 2008 ▶); software used to prepare material for publication: *SHELXTL*.

## Supplementary Material

Crystal structure: contains datablocks I, global. DOI: 10.1107/S1600536810037141/is2575sup1.cif

Structure factors: contains datablocks I. DOI: 10.1107/S1600536810037141/is2575Isup2.hkl

Additional supplementary materials:  crystallographic information; 3D view; checkCIF report

## Figures and Tables

**Fig. 1.**
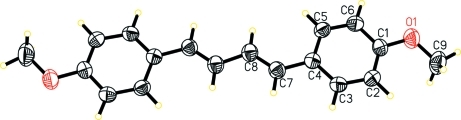
The molecular structure of (I), with atom labels and 50% probability displacement ellipsoids for non-H atoms.

**Fig. 2.**
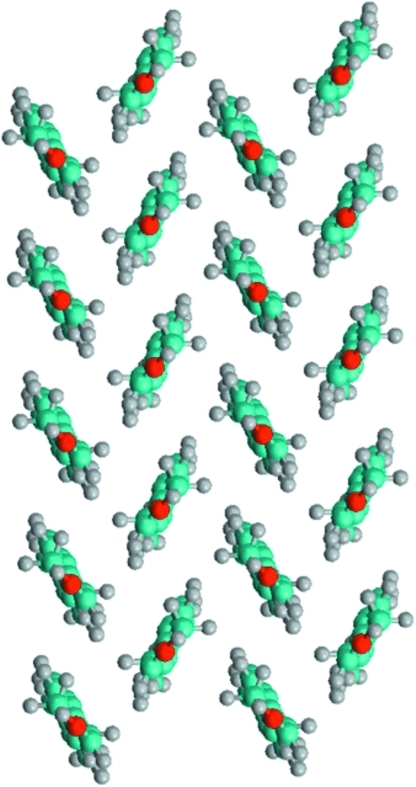
The herringbone packing of (I) in the crystal structure.

## supplementary crystallographic information

### Comment

Although the crystal structures of a number of butadiene molecules have been reported (George *et al.*, 1998; Vishnumurthy *et al.*, 2002; Davis *et al.*, 2004, 2008; Kumar *et al.*, 2009; Ono *et al.*, 2009), that of the title compound, C_18_H_18_O_2_, (I), has not been determined and the structure is reported here (Fig. 1). There are four molecules of (I) per unit cell. The symmetrical molecules are arranged in a herringbone fashion (Koren *et al.*, 2003) in which the molecules are packed in an edge-to-face orientation (Fig. 2).

Thermal properties: On heating, crystals of (I) melted at 237 °C, which on further heating sublimed at 246 °C. The sublimed-condensed crystals were chemically unaltered as evidenced by NMR and MS analyses.

### Experimental

A mixture of diethyl-4-methoxybenzylphosphonate (1 equiv) and potassium *tert*-butoxide (5 equiv) were stirred in dry DMF at room temperature and cooled to 273 K. 4-Methoxycinnamaldehyde (1 equiv) dissolved in dry DMF was slowly added into the solution. The reaction mixture was allowed to stir for 12 h at room temperature. TLC analysis indicated completion of reaction. Reaction mixture was poured into ice water, extracted with dichloromethane and concentrated under reduced pressure. The residue was washed with ethyl acetate and filtered. The compound being insoluble in ethyl acetate remained in the residue. This was repeatedly washed with ethyl acetate (small quantities) to obtain pure title compound. The small amount of compound which remained in the filtrate was recovered by column chromatography through silica gel (100–200 mesh), using 5% ethyl acetate/hexane as the mobile phase. Single crystals obtained from ethylacetate at room temperature were of poor quality (high *R* value) and the structure determination was carried out at 100 K. Fresh crystals were grown from chloroform/hexanes at room temperature which were of higher quality to permit X-ray analysis at 293 K. The data presented herein are from the latter determination.

### Refinement

H atoms bonded to N and O atoms were located in a difference map and refined with distance restraints of O—H = 0.84 (2) and N—H = 0.87 (2) Å, and with *U*_iso_(H) = 1.2*U*_eq_(N,O). Other H atoms were positioned geometrically and refined using a riding model (including free rotation about the ethanol C—C bond), with C—H = 0.95–0.99 Å and with *U*_iso_(H) = 1.2 (1.5 for methyl groups) times *U*_eq_(C).

### Crystal data

**Table:**

C_18_H_18_O_2_	*F*(000) = 568
*M**_r_* = 266.32	*D*_x_ = 1.224 Mg m^−^^3^
Orthorhombic, *P**b**c**a*	Mo *K*α radiation, λ = 0.71073 Å
Hall symbol: -P 2ac 2ab	Cell parameters from 7512 reflections
*a* = 7.3543 (3) Å	θ = 2.6–23.6°
*b* = 6.2617 (3) Å	µ = 0.08 mm^−^^1^
*c* = 31.3872 (13) Å	*T* = 293 K
*V* = 1445.39 (11) Å^3^	Pyramidal, colourless
*Z* = 4	0.25 × 0.22 × 0.22 mm

### Data collection

**Table:**

Bruker X8 APEXII CCD area-detector diffractometer	1658 independent reflections
Radiation source: fine-focus sealed tube	1287 reflections with *I* > 2σ(*I*)
graphite	*R*_int_ = 0.036
φ and ω scans	θ_max_ = 27.5°, θ_min_ = 1.3°
Absorption correction: numerical (*SADABS*; Sheldrick, 2006)	*h* = −8→9
*T*_min_ = 0.981, *T*_max_ = 0.983	*k* = −8→8
40427 measured reflections	*l* = −40→37

### Refinement

**Table:**

Refinement on *F*^2^	Primary atom site location: structure-invariant direct methods
Least-squares matrix: full	Secondary atom site location: difference Fourier map
*R*[*F*^2^ > 2σ(*F*^2^)] = 0.045	Hydrogen site location: inferred from neighbouring sites
*wR*(*F*^2^) = 0.110	H-atom parameters constrained
*S* = 1.08	*w* = 1/[σ^2^(*F*_o_^2^) + (0.0429*P*)^2^ + 0.3178*P*] where *P* = (*F*_o_^2^ + 2*F*_c_^2^)/3
1658 reflections	(Δ/σ)_max_ < 0.001
92 parameters	Δρ_max_ = 0.13 e Å^−^^3^
0 restraints	Δρ_min_ = −0.13 e Å^−^^3^

### Special details

**Table:**

Experimental. 2010–02-01 # Formatted by publCIF
Geometry. All e.s.d.'s (except the e.s.d. in the dihedral angle between two l.s. planes) are estimated using the full covariance matrix. The cell e.s.d.'s are taken into account individually in the estimation of e.s.d.'s in distances, angles and torsion angles; correlations between e.s.d.'s in cell parameters are only used when they are defined by crystal symmetry. An approximate (isotropic) treatment of cell e.s.d.'s is used for estimating e.s.d.'s involving l.s. planes.
Refinement. Refinement of *F*^2^ against ALL reflections. The weighted *R*-factor *wR* and goodness of fit *S* are based on *F*^2^, conventional *R*-factors *R* are based on *F*, with *F* set to zero for negative *F*^2^. The threshold expression of *F*^2^ > σ(*F*^2^) is used only for calculating *R*-factors(gt) *etc*. and is not relevant to the choice of reflections for refinement. *R*-factors based on *F*^2^ are statistically about twice as large as those based on *F*, and *R*- factors based on ALL data will be even larger.

### Fractional atomic coordinates and isotropic or equivalent isotropic displacement parameters (Å^2^)

**Table:**

	*x*	*y*	*z*	*U*_iso_*/*U*_eq_	
O1	−0.01884 (15)	0.42509 (16)	0.19955 (3)	0.0584 (3)	
C1	−0.00667 (17)	0.4944 (2)	0.15818 (4)	0.0420 (3)	
C2	0.07908 (18)	0.3855 (2)	0.12580 (4)	0.0458 (3)	
H2	0.1341	0.2543	0.1310	0.055*	
C3	0.08211 (18)	0.4740 (2)	0.08547 (4)	0.0441 (3)	
H3	0.1386	0.3988	0.0636	0.053*	
C4	0.00422 (16)	0.6707 (2)	0.07623 (4)	0.0396 (3)	
C5	−0.08465 (17)	0.7752 (2)	0.10963 (4)	0.0440 (3)	
H5	−0.1411	0.9056	0.1046	0.053*	
C6	−0.09002 (18)	0.6884 (2)	0.14980 (4)	0.0455 (3)	
H6	−0.1501	0.7605	0.1716	0.055*	
C7	0.02594 (18)	0.7620 (2)	0.03382 (4)	0.0452 (3)	
H7	0.0769	0.6729	0.0133	0.054*	
C8	−0.01863 (18)	0.9584 (2)	0.02086 (4)	0.0461 (3)	
H8	−0.0777	1.0472	0.0402	0.055*	
C9	0.0738 (3)	0.2339 (3)	0.21039 (5)	0.0789 (6)	
H9A	0.0254	0.1176	0.1940	0.118*	
H9B	0.0577	0.2050	0.2402	0.118*	
H9C	0.2010	0.2497	0.2043	0.118*	

### Atomic displacement parameters (Å^2^)

**Table:**

	*U*^11^	*U*^22^	*U*^33^	*U*^12^	*U*^13^	*U*^23^
O1	0.0773 (7)	0.0568 (6)	0.0410 (6)	0.0056 (6)	0.0018 (5)	0.0061 (5)
C1	0.0447 (7)	0.0442 (7)	0.0371 (7)	−0.0047 (6)	−0.0013 (5)	0.0012 (6)
C2	0.0485 (8)	0.0395 (7)	0.0495 (8)	0.0068 (6)	0.0004 (6)	0.0015 (6)
C3	0.0454 (7)	0.0431 (7)	0.0439 (7)	0.0041 (6)	0.0049 (6)	−0.0034 (6)
C4	0.0366 (6)	0.0405 (7)	0.0416 (7)	−0.0029 (5)	−0.0018 (5)	−0.0007 (5)
C5	0.0446 (7)	0.0395 (7)	0.0479 (8)	0.0056 (6)	0.0001 (6)	−0.0005 (6)
C6	0.0485 (8)	0.0439 (7)	0.0441 (7)	0.0043 (6)	0.0036 (6)	−0.0047 (6)
C7	0.0464 (7)	0.0484 (8)	0.0407 (7)	−0.0010 (6)	−0.0012 (6)	−0.0009 (6)
C8	0.0463 (7)	0.0488 (8)	0.0430 (7)	−0.0031 (6)	−0.0036 (6)	0.0015 (6)
C9	0.1102 (16)	0.0692 (11)	0.0574 (10)	0.0158 (11)	−0.0010 (10)	0.0204 (9)

### Geometric parameters (Å, °)

**Table:**

O1—C1	1.3721 (15)	C5—C6	1.3735 (18)
O1—C9	1.4190 (19)	C5—H5	0.9300
C1—C2	1.3768 (18)	C6—H6	0.9300
C1—C6	1.3860 (18)	C7—C8	1.3367 (19)
C2—C3	1.3820 (18)	C7—H7	0.9300
C2—H2	0.9300	C8—C8^i^	1.435 (3)
C3—C4	1.3890 (18)	C8—H8	0.9300
C3—H3	0.9300	C9—H9A	0.9600
C4—C5	1.3978 (18)	C9—H9B	0.9600
C4—C7	1.4573 (18)	C9—H9C	0.9600
			
C1—O1—C9	117.55 (12)	C5—C6—C1	120.54 (12)
O1—C1—C2	124.87 (12)	C5—C6—H6	119.7
O1—C1—C6	115.34 (12)	C1—C6—H6	119.7
C2—C1—C6	119.78 (12)	C8—C7—C4	127.73 (13)
C1—C2—C3	119.00 (12)	C8—C7—H7	116.1
C1—C2—H2	120.5	C4—C7—H7	116.1
C3—C2—H2	120.5	C7—C8—C8^i^	124.37 (17)
C2—C3—C4	122.70 (12)	C7—C8—H8	117.8
C2—C3—H3	118.6	C8^i^—C8—H8	117.8
C4—C3—H3	118.6	O1—C9—H9A	109.5
C3—C4—C5	116.82 (12)	O1—C9—H9B	109.5
C3—C4—C7	119.55 (12)	H9A—C9—H9B	109.5
C5—C4—C7	123.56 (12)	O1—C9—H9C	109.5
C6—C5—C4	121.12 (12)	H9A—C9—H9C	109.5
C6—C5—H5	119.4	H9B—C9—H9C	109.5
C4—C5—H5	119.4		
			
C9—O1—C1—C2	4.3 (2)	C7—C4—C5—C6	−175.32 (12)
C9—O1—C1—C6	−176.19 (14)	C4—C5—C6—C1	0.1 (2)
O1—C1—C2—C3	−179.72 (12)	O1—C1—C6—C5	179.15 (12)
C6—C1—C2—C3	0.81 (19)	C2—C1—C6—C5	−1.33 (19)
C1—C2—C3—C4	0.9 (2)	C3—C4—C7—C8	−170.76 (13)
C2—C3—C4—C5	−2.09 (19)	C5—C4—C7—C8	6.0 (2)
C2—C3—C4—C7	174.90 (13)	C4—C7—C8—C8^i^	175.49 (15)
C3—C4—C5—C6	1.54 (18)		

Symmetry codes: (i) −*x*, −*y*+2, −*z*.
